# Release of HMGB1 and Toll-like Receptors 2, 4, and 9 Signaling Are Modulated by *Bifidobacterium animalis* subsp. *lactis* BB-12 and *Salmonella* Typhimurium in a Gnotobiotic Piglet Model of Preterm Infants

**DOI:** 10.3390/ijms24032329

**Published:** 2023-01-24

**Authors:** Igor Splichal, Sharon M. Donovan, Zdislava Kindlova, Zbynek Stranak, Vera Neuzil Bunesova, Marek Sinkora, Katerina Polakova, Barbora Valaskova, Alla Splichalova

**Affiliations:** 1 Laboratory of Gnotobiology, Institute of Microbiology, Czech Academy of Sciences, 549 22 Novy Hradek, Czech Republic; 2 Department of Food Science and Human Nutrition, University of Illinois, Urbana, IL 61801, USA; 3 Department of Neonatology, Institute for the Care of Mother and Child, 147 00 Prague, Czech Republic; 4 Department of Microbiology, Nutrition and Dietetics, Czech University of Life Sciences in Prague, 160 00 Prague, Czech Republic

**Keywords:** *Bifidobacterium animalis* subsp. *lactis* BB-12, *Salmonella* Typhimurium, high mobility group box 1, Toll-like receptors, tight junction proteins, mucin, intestinal barrier, inflammatory cytokines, immunodeficient host

## Abstract

Gnotobiotic (GN) animals with defined microbiota allow us to study host–microbiota and microbiota–microbiota interferences. Preterm germ-free (GF) piglets were mono-associated with probiotic *Bifidobacterium animalis* subsp. *lactis* BB-12 (BB12) to ameliorate/prevent the consequences of infection with the *Salmonella* Typhimurium strain LT2 (LT2). Goblet cell density; expression of Toll-like receptors (TLRs) 2, 4, and 9; high mobility group box 1 (HMGB1); interleukin (IL)-6; and IL-12/23p40 were analyzed to evaluate the possible modulatory effect of BB12. BB12 prevented an LT2-induced decrease of goblet cell density in the colon. TLRs signaling modified by LT2 was not influenced by the previous association with BB12. The expression of HMGB1, IL-6, and IL12/23p40 in the jejunum, ileum, and colon and their levels in plasma were all decreased by BB12, but these changes were not statistically significant. In the colon, differences in HMGB1 distribution between the GF and LT2 piglet groups were observed. In conclusion, the mono-association of GF piglets with BB12 prior to LT2 infection partially ameliorated the inflammatory response to LT2 infection.

## 1. Introduction

Preterm birth (PTB) is a birth that occurs before 37 weeks of gestation, and its incidence affects about 11% of pregnancies [1]. Various reasons can trigger PTB, and the inflammatory process is one of them [2]. In addition, preterm infants have a low birth weight and underdeveloped organ systems, making them more susceptible to many life-threatening comorbidities [2]. These factors and their possible concurrence result in special requirements for preterm infants that need supportive care in the neonatal intensive care unit (NICU) [3]. However, despite this particular regimen, the preterm infant suffers from increased morbidity that is inversely proportional to the length of gestation [4].

The initial colonization of vaginally born infants occurs immediately during childbirth by the mother’s vaginal and fecal microbiota. This settlement starts with pioneer settlers that create suitable conditions for their followers [5]. However, colonization occurs more rapidly than was believed [6], because strictly anaerobic bifidobacteria are present in the feces of some infants on the first day after birth [7]. The establishment of balanced microbiota (eubiosis) in newborns is negatively influenced by antibiotic treatment of the mother or newborn and formula feeding [8]. Cesarean-delivered infants are not exposed to the mother’s vaginal and fecal microbiota and are, instead, colonized with microbes from the surgery room and NICU surroundings, which can have competitive advantages for their antibiotic resistance [9] and can cause nosocomial infections. Furthermore, this microbiota with low diversity allows the overgrowth of pathobionts that are usually suppressed in their growth [10]. Thus, the early establishment of a balanced microbiota is crucial and beneficial for the host’s development and health [11]. For this reason, it is necessary to pay great attention to the initial colonization that will impact short- and long-term health [12].

Microbes express pathogen-associated molecular patterns (PAMPs) that are [13] recognized by pattern-recognition receptors (PRRs) to trigger an immune defense response. One of the beneficial effects of the gastrointestinal (GI) microbiota on neonatal hosts is the stimulation of immune system development [14]. In contrast to PAMPs, damage-associated molecular patterns (DAMPs) are molecules produced by the host after stimulation or cellular damage [15] that are usually hidden from immune recognition. Both PAMPs and DAMPs represent danger signals, are sensed by PRRs, and induce inflammatory reactions to maintain homeostasis [16]. Toll-like receptors (TLRs) are PRRs that recognize both PAMPs and DAMPs [15,17]. TLRs sense various bacterial motifs, such as lipoproteins, lipoteichoic acid, peptidoglycan (TLR2), lipopolysaccharide (LPS; TLR4), and CpG (TLR9) [13]. TLR2, TLR4, and TLR9 also sense the DAMPs, high mobility group 1 (HMGB1) [18].

Probiotics are live microorganisms that beneficially affect the host’s health. The treatment of preterm infants with probiotics positively influenced the GI microbial ecosystem and showed preventive effects against the development of necrotizing enterocolitis (NEC) [19] and sepsis [20]. Primary colonization of the preterm infant GI with probiotics supported further colonization with probiotic bacteria [21]. Bifidobacteria belong to the first colonizers and principal inhabitants of the infant’s intestine [7]. Together with lactobacilli, they form the main components of probiotic preparations [22]. Probiotic persistence among indigenous microbiota is usually transient and depends on GI microbiota composition [23]. Thus, the newborn GI tract with no fully established balanced microbiota and low colonization resistance [24] suggests an opportunity for longer-time colonization with probiotics and defined microbiota [23]. *Bifidobacterium animalis* subsp. *lactis* BB-12 (BB12) is a widely used bifidobacterial probiotic strain with excellent gastric acid and bile tolerance and strong mucus-adherence properties [25]. BB12 showed its ability to reduce *Salmonella* growth in the GIT of mice and alleviate the consequences of the infection [26].

Gnotobiotic (GN) animals are microbiologically defined animals consisting of germ-free (GF) animals and animals associated with simple defined microbiota [27]. The GF animals with absent microbiota show lower colonization resistance and higher sensitivity to enteric infections [28]. They are suitable animal models for studies of host–bacteria and bacteria–bacteria interferences. Our study aimed to evaluate the possibility of modulating TLRs signaling by administration of a widely used probiotic bacteria to alleviate the consequences of enteric infections. HMGB1, a marker of the severity of enteric infection and sepsis, and a potent inflammatory inducer, was used as the main indicator molecule to evaluate the inflammatory process. Thus, we studied the direct interactions between *Bifidobacterium animalis* subsp. *lactis* BB-12 (BB12) and *Salmonella* Typhimurium LT2 (LT2) in a GN piglet model of preterm infants [29]. The preterm GF piglets were associated with *B. animalis* BB-12 (BB12) for one week prior to being infected with enteric pathogen *S*. Typhimurium LT2 (BB12 + LT2) or infected with *S*. Typhimurium LT2 alone (LT2).

## 2. Results

### 2.1. Clinical Signs of Enterocolitis

The non-infected piglets (GF and BB12 groups) did not show any signs of enterocolitis. In contrast, the Salmonella-infected piglets (LT2) were sleepy and had anorexia with non-bloody diarrhea and fever. The piglets associated with BB12 and one-week later infected with LT2 (BB12 + LT2) showed milder diarrhea than the piglets infected with LT2 only.

### 2.2. Goblet Cells in the Ileum

**Figure 1:**
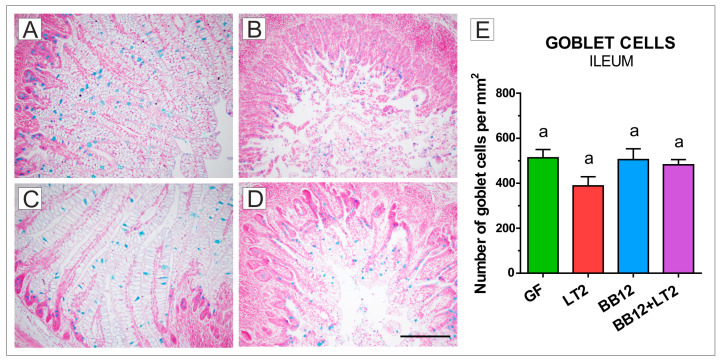
Goblet cells (blue color) in the ileum of gnotobiotic piglets. Number of goblet cells per mm^2^ in the ileum of the one-week-old piglets: germ-free (GF; (**A**)), infected with *S*. Typhimurium LT2 for 24 h (LT2; (**B**)), associated with *B. animalis* subsp. *lactis* BB-12 (BB12; (**C**)), and associated with BB12 and infected with LT2 for 24 h (BB12 + LT2; (**D**)). Six samples from each group were analyzed, and statistical differences were calculated by a two-way ANOVA with Tukey’s multiple comparison post hoc test. The values are presented as mean + SEM, and *p* < 0.05 among groups are denoted by different letters above the columns (**E**). A scale bar (**D**) depicts 200 μm.

The GF (Figure 1A) and BB12 (Figure 1C) piglets showed long villi with many vacuolated enterocytes and mucin-producing, blue-stained goblet cells among enterocytes. Piglets infected with *S*. Typhimurium (Figure 1B) had shortened and damaged villi with desquamated epithelial cells in the lumen. Colonization with BB12 did not fully protect ileal villus morphology against injury induced by *S*. Typhimurium infection (Figure 1D). The number of goblet cells was reduced in the LT2 group, but this decrease was not statistically significant (Figure 1E).

### 2.3. Goblet Cells in the Colon

**Figure 2:**
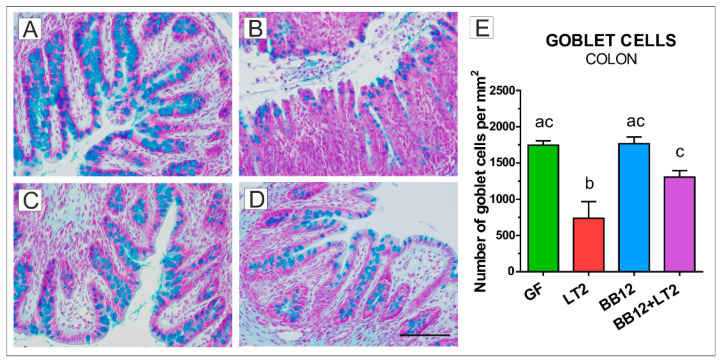
Goblet cells (blue color) in the colon of gnotobiotic piglets. Number of goblet cells per mm^2^ in the colon of the one-week-old gnotobiotic piglets: germ-free (GF; (**A**)), infected with *S*. Typhimurium LT2 for 24 h (LT2; (**B**)), associated with *B. animalis* subsp. *lactis* BB-12 (BB12; (**C**)), and associated with BB12 and infected with LT2 for 24 h (BB12 + LT2; (**D**)). Six samples from each group were analyzed, and statistical differences were calculated by a two-way ANOVA with Tukey’s multiple comparison post hoc test. The values are presented as mean + SEM, and a *p* < 0.05 among groups is denoted by different letters above the columns (**E**). A scale bar (**D**) depicts 100 μm.

*S*. Typhimurium infection significantly reduced the number of goblet cells in the colon in the LT2 groups (Figure 2B,E) compared to the other groups (Figure 2A,C–E). Prior association with BB12 (BB12 + LT2; Figure 2C) prevented a decrease in the goblet cell counts, as is comparable to the GF (Figure 2A,E), BB12 (Figure 2C,E), and BB12 + LT2 (Figure 2D,E) piglets.

### 2.4. TLR2, TLR4, TLR9, MyD88, TRIF, and RAGE mRNA in Ileum

**Figure 3:**
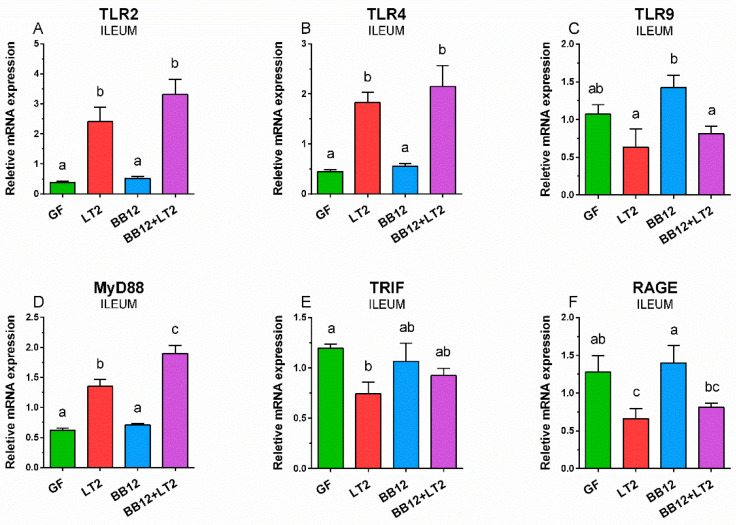
Relative expression (fold-change) of TLR2 (**A**), TLR4 (**B**), TLR9 (**C**), MyD88 (**D**), TRIF (**E**), and RAGE (**F**) mRNA in the ileum of the one-week-old gnotobiotic piglets: germ-free (GF), infected with *S*. Typhimurium LT2 for 24 h (LT2), associated with *B. animalis* subsp. *lactis* BB-12 (BB12), and associated with BB12 and infected with LT2 for 24 h (BB12 + LT2). Six samples from each group were analyzed, and statistical differences were calculated by a two-way ANOVA with Tukey’s multiple comparison post hoc test. The values are presented as mean + SEM, and a *p* < 0.05 among groups is denoted by different letters above the columns.

BB12 did not increase the expression of TLR2 mRNA in the ileum compared to the GF piglet control (Figure 3A). In contrast, both groups infected with *S*. Typhimurium (LT2 and BB12 + LT2) had significantly higher expression. The presence of BB12 (BB12 + LT2) did not significantly influence the TLR2 expression compared to *S*. Typhimurium infection alone (LT2). Similar changes of mRNA expression were found in TLR4 (Figure 3B). An opposite trend was found in TLR9 mRNA expression (Figure 3C). TLR9 mRNA expression was comparable in GF and BB12 groups but was downregulated by *Salmonella* infection. The prior association with BB12 (BB12 + LT2) did not ameliorate the LT2-inducted downregulation of TLR-9 expression. BB12 did not influence MyD88 mRNA expression, but it was significantly upregulated by *Salmonella* (Figure 3D). The presence of BB12 enhanced this expression, so the expression in BB12 + LT2 group was significantly higher than in the LT2 group. In contrast, *Salmonella* downregulated the expression of TRIF mRNA (Figure 3E). *Salmonella* infection downregulated RAGE mRNA expression compared to GF, but this downregulation was only significant for LT2, and not for BB12 + LT2 (Figure 3F).

### 2.5. TLR2, TLR4, TLR9, MyD88, TRIF, and RAGE mRNA in Colon

**Figure 4:**
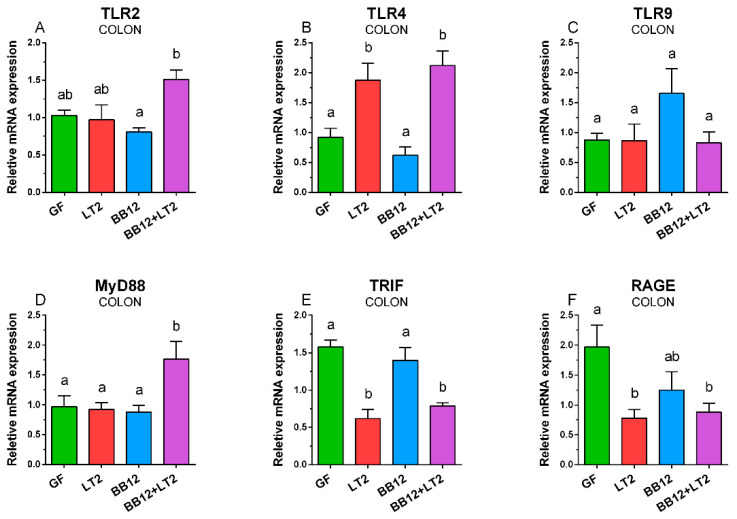
Relative expression (fold-change) of TLR2 (**A**), TLR4 (**B**), TLR9 (**C**), MyD88 (**D**), TRIF (**E**), and RAGE (**F**) mRNA in the colon of the one-week-old gnotobiotic piglets: germ-free (GF), infected with *S*. Typhimurium LT2 for 24 h (LT2), associated with *B. animalis* subsp. *lactis* BB-12 (BB12), and associated with BB12 and infected with LT2 for 24 h (BB12 + LT2). Six samples from each group were analyzed, and statistical differences were calculated by a two-way ANOVA with Tukey’s multiple comparison post hoc test. The values are presented as mean + SEM, and a *p* < 0.05 among groups is denoted by different letters above the columns.

BB12 downregulated TLR2 mRNA expression in the colon, but this downregulation in comparison to GF and LT2 groups was statistically non-significant (Figure 4A). However, a previous association with BB12 (BB12 + LT2) caused significant upregulation in comparison to the BB12 group alone. Simultaneously, this upregulation was not statistically significant in comparison to the GF and LT2 groups. *Salmonella* significantly upregulated TLR4 mRNA expression in both infected groups (LT2 and BB12 + LT2) (Figure 4B). TLR9 mRNA expression was comparable among all groups (Figure 4C). MyD88 mRNA was significantly upregulated in the BB12 + LT2 group only (Figure 4D). TRIF mRNA was significantly downregulated in both *Salmonella*-infected groups compared to both non-infected groups (Figure 4E). RAGE mRNA expression was downregulated in the *Salmonella*-infected groups (LT2 and BB12 + LT2) (Figure 4F).

### 2.6. TLR2, TLR4, TLR9, MyD88, TRIF, and RAGE mRNA in Mesenteric Lymph Nodes

**Figure 5:**
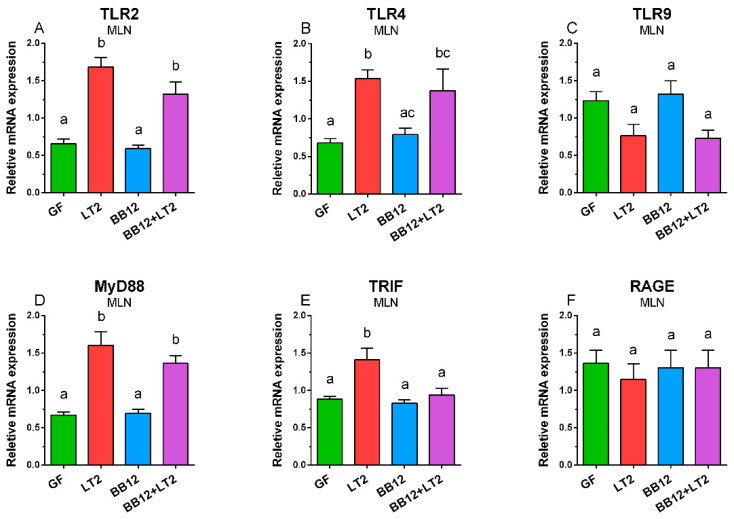
Relative expression (fold-change) of TLR2 (**A**), TLR4 (**B**), TLR9 (**C**), MyD88 (**D**), TRIF (**E**), and RAGE (**F**) mRNA in the mesenteric lymph nodes of the one-week-old piglets: germ-free (GF), infected with *S*. Typhimurium LT2 for 24 h (LT2), associated with *B. animalis* subsp. *lactis* BB-12 (BB12), and associated with BB12 and infected with LT2 for 24 h (BB12 + LT2). Six samples from each group were analyzed, and statistical differences were calculated by a two-way ANOVA with Tukey’s multiple comparison post hoc test. The values are presented as mean + SEM, and a *p* < 0.05 among groups is denoted by different letters above the columns.

*Salmonella* significantly upregulated TLR2 mRNA expression in MLN (Figure 5A). The same trend was observed for TLR4 mRNA, but only induction in the LT2-infected groups (LT2 and BB12) was significant against the GF group only (Figure 5B). The opposite trend was found for TLR9, but the suppression by *Salmonella* was not statistically significant (Figure 5C). MyD88 mRNA was significantly induced by *Salmonella* in both infected groups (Figure 5D), but in the case of TRIF mRNA, this significant upregulation was observed in the LT2 group only (Figure 5E). Finally, no effect of LT2 or BB12 on RAGE mRNA expression in MLN was found (Figure 5F).

### 2.7. HMGB1 Expression in the Colon

**Figure 6:**
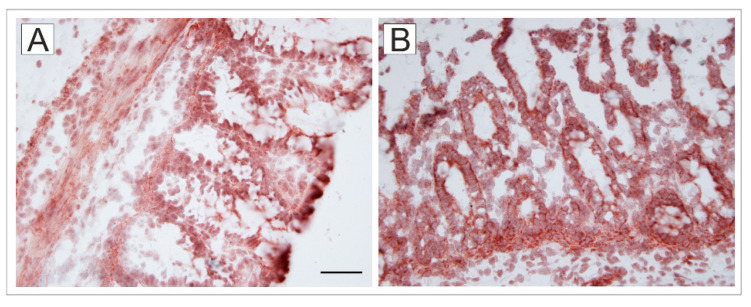
Expression of HMGB1 in the colon. Representative micrographs of the germ-free (GF; (**A**)) and *S*. Typhimurium LT2-infected piglets for 24 h (LT2; (**B**)) are depicted. The scale bar (**A**) corresponds to 50 μm.

In the colon of GF piglets (Figure 6A), the nuclear protein HMGB1 was localized in both the nucleus and the cytoplasm, whereas in LT2 piglets (Figure 6B), HMGB1 was localized mainly in the cytoplasm.

### 2.8. Intestinal Levels of HMGB1, IL-6, and IL-12/23p40

**Figure 7:**
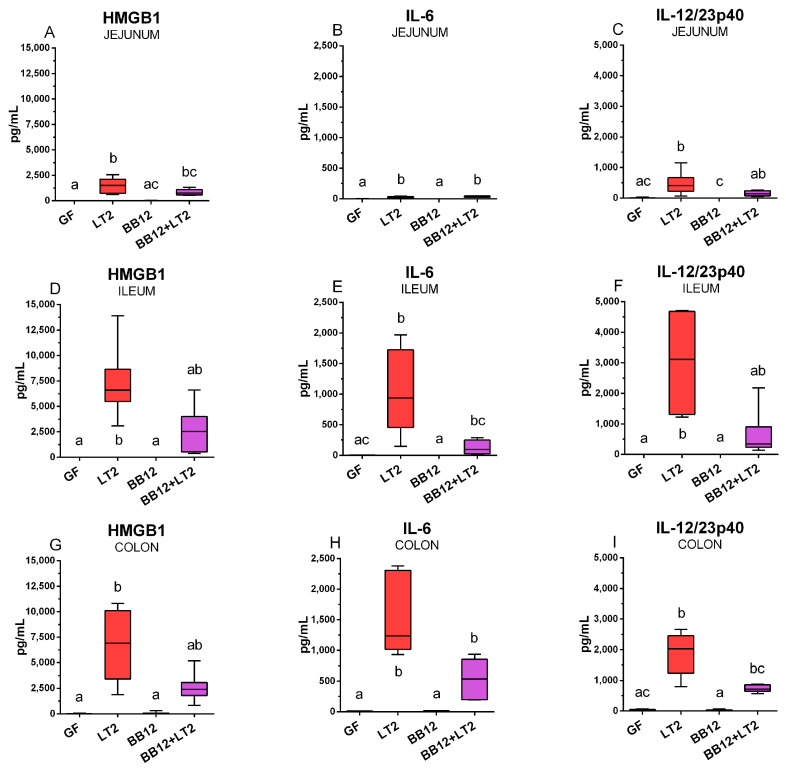
Abundance of HMGB1 (**A**,**D**,**G**), IL-6 (**B**,**E**,**H**), and IL-12/23p40 (**C**,**F**,**I**) proteins in the jejunum (**A**–**C**), ileum (**D**–**F**), and colon (**G**–**I**) of the one-week-old piglets: germ-free (GF), infected with *S*. Typhimurium LT2 for 24 h (LT2), associated with *B. animalis* subsp. *lactis* BB-12 (BB12), and associated with BB12 and infected with LT2 for 24 h (BB12 + LT2). Six samples from each group were analyzed, and statistical differences were calculated by the Kruskal–Wallis test with Dunn’s multiple comparison post hoc test. The values are presented as boxes and whiskers indicating the lower and upper quartiles, the central line is the median, and the ends of the whiskers depict the minimal and maximal values. A *p* < 0.05 among groups is denoted with different letters around the columns.

HMGB1 release was significantly induced in the jejunum with *Salmonella* infection (Figure 7A) compared to the GF group. The association with BB12 did not induce HMGB1 release in the jejunum. The previous association of the piglets with BB12 in the (BB12 + LT2) group prevented the significant induction of HMGB1 (Figure 7A). However, differences between LT2 and BB12 + LT2 were non-significant. The IL-6 levels in the *Salmonella*-infected piglets were significantly increased (Figure 7B). IL-12/23p40 was also induced by the infection (Figure 7C). The previous association of piglets with BB12 significantly suppressed this increase.

In the ileum, HMGB1 levels showed a similar trend as in the jejunum; that is, they were significantly induced in the LT2 group and previously associated with BB12 non-significantly reduced this increase (Figure 7D). The IL-6 levels in the ileum were upregulated by the *Salmonella* infection, but suppression by the previous association with BB12 resulted in a non-significant increase against the control GF group (Figure 7E). At the same time, the suppression was not statistically significant compared to that of the LT2 group. A similar trend of induction/suppression in the ileum was also found in IL-12/23p40 group (Figure 7F).

As was observed in the jejunum and ileum, HMGB1 BB12 suppressed HMGB1 concentrations in the colon (Figure 7G), but the difference was not statistically significant. IL-6 levels were significantly induced by the infection with *Salmonella* (Figure 7H). The previously applied BB12 non-significantly suppressed IL-12/23p40 levels in the colon (Figure 7I).

All biomarkers showed low levels in the jejunum (Figure 7A–C). They were highly increased in the ileum (Figure 7D–F) and colon (Figure 7G–I) of both LT2-infected groups, and these values were comparable in both organs. The same ratio of the *y*-axis in individual biomarkers was used to clearly depict this trend throughout the intestine.

### 2.9. HMGB, IL-6, and IL-12/23p40 in Plasma

**Figure 8:**
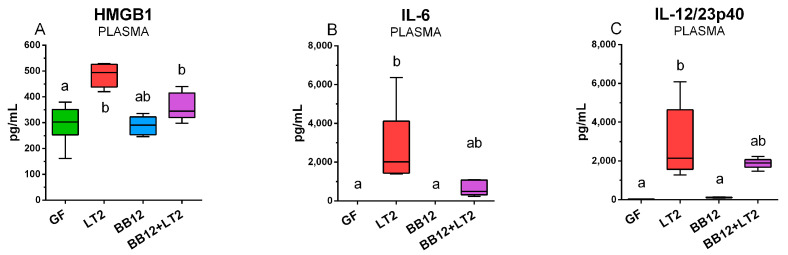
Plasma concentrations of HMGB1 (**A**), IL-6 (**B**) and IL-12/23p40 (**C**) proteins of one-week-old piglets: germ-free (GF), infected with *S*. Typhimurium LT2 for 24 h (LT2), associated with *B. animalis* subsp. *lactis* BB12 (BB12), and associated with BB12 and infected with LT2 for 24 h (BB12 + LT2). Six samples in each group were analyzed and statistical differences were calculated by the Kruskal–Wallis test with Dunn’s multiple comparison post hoc test. The values are presented as boxes and whiskers indicating the lower and upper quartiles, the central line is the median, and the ends of the whiskers depict the minimal and maximal values. A *p* < 0.05 among groups is denoted with different letters around the columns.

The levels of plasmatic HMGB1 (Figure 8A), IL-6 (Figure 8B), and IL-12/23p40 (Figure 8C) reflect the situation in the intestine. It means that *Salmonella* induced these levels, and the previous association with BB12 suppressed them, but this suppression was not statistically significant.

## 3. Discussion

### 3.1. Animal Models of Immunocompromised Host and Probiotics

Most reports refer to probiotics as beneficial and safe for preterm infants [23]. However, it is necessary to consider that preterm neonates have underdeveloped immune systems and compromised intestinal barrier integrity [30]. It simplifies bacterial translocation, and these infants are at increased risk of probiotic-caused sepsis [31]. The experimental work with human volunteers is limited [32], and neither two-dimensional (2D) nor three-dimensional (3D; organoids) cell culture systems [33] sufficiently simulate a complex reaction of the whole organism. Thus, suitable translational animal models play a vital role in human disease research [34,35,36,37].

### 3.2. Gnotobiotic Piglet Translational Model

The similarities in anatomy, physiology, genetics, immunology [38], and microbiome composition [39,40] predestine pigs as animal models of human diseases. Pig translational models are used for studies in nutrition and gastroenterology [41], infectious diseases [42], and sepsis [35]. The potential of the pig as an organ donor for humans deepens the attraction of this animal model [43]. Several research groups studied bacterial translocation and sepsis [44,45,46,47], the ontogeny of innate [29,45] and adaptive [48,49] immunity, and NEC [36,50,51] on preterm piglets.

In our experiments, we infected the one-week-old preterm GF piglets with *S*. Typhimurium strain LT2 for 24 h [46]. This *Salmonella* strain was avirulent for one-week-old conventional (CV) piglets [52] but lethal for term GF piglets, which died 36–48 h post-infection [53]. Thus, its virulence is influenced by the presence of a microbiota and, expectedly, also the microbiome composition. Moreover, the virulence of the *S*. Typhimurium serovars for the GF piglets depends on the form completeness of its LPS. For example, Gram-negative bacteria secrete smooth LPS chemotype (S-LPS), which is more virulent than rough (R-LPS) chemotype mutants [47,53]. Colonization resistance, presence of maternal immunoglobulins and immune cells [54], and stimulation of innate immunity [55] in the CV piglets are probably responsible for their resistance to LT2 infection [9,13,29,34,52,56,57].

### 3.3. Intestinal Barrier

A single epithelial cell layer creates a specific barrier between the bacteria-rich intestinal lumen and the host’s organism. Adjacent enterocytes are joined in their apical part with tight junction proteins, e.g., claudins and occludin, and create a semipermeable interface that protects the host against penetration of harmful dietary antigens and invading pathogens and their toxins [58]. The epithelial layer is covered with mucin composed of a lumen-oriented movable upper layer and an enterocyte-touched fixed lower layer [59]. The disruption of the mucus layer causes intestinal inflammation and facilitates bacterial translocation [60,61]. Mucins are produced and secreted from goblet cells which are specialized enterocytes and colonocytes in the small intestine and colon, respectively [58]. The main intestinal mucin in humans and mice is acid mucin 2 (MUC2) [59]. An impaired MUC2 synthesis predisposed preterm CV piglets to develop necrotizing enterocolitis [62], and a defect in the production of MUC2 dramatically increased the sensitivity of mice to infection with S. Typhimurium [63]. The presence of bacteria stimulates the production of mucin and GF animals show thinner mucin layer compare to CV ones [58].

We measured acid mucin-producing goblet cell density in the ileum and colon of the preterm GN piglets. In our previous study with the term piglets [61], we found that the term GN piglets showed a higher number of goblet cells in the ileum. In the current study, preterm GN piglets had a comparable goblet cell density in the ileum and colon. In contrast, comparable numbers of the acid and neutral mucin-containing goblet cells in the distal small intestine, but lower in the colon, were found in the preterm versus term CV piglets [61,64,65]. Mucin degradation allows for easier penetration of harmful bacteria, and the absence of mucin-degrading activities is a safety criterion for probiotic candidates [63,66].

In previous studies, BB12 alone or in combination with LT2 did not weaken the intestinal barrier or increase bacterial translocation [46], as was shown in mucinolytic B. boum RP36 [61]. Thus, we believe that BB12 did not provoke an adverse effect in the ileum of the preterm GN piglets. In the colon of the term GN piglets, which has a higher goblet cell density than the ileum, a negative effect of Salmonella was shown without an influence of either B. boum strain studied [61]. This significant Salmonella-induced downregulation of goblet cell density in the colon was also found in preterm piglets; however, this effect was ameliorated by probiotic BB12, supporting a beneficial effect on the host. Differences in goblet cell count in piglets can also be influenced by the formula used, as was found in formula-fed compared to colostrum-fed CV preterm piglets [62]. In our experiment, all GN piglet groups were fed the same way (Splichalova et al., 2018), excluding the possible effect of the diet.

### 3.4. Receptors and Biomarkers

TLRs 2, 4, and 9 are commonly classified as Gram-positive (TLR2), Gram-negative (TLR4), and pan-bacteria (TLR9) recognizing receptors [67]. However, they are not narrowly specific to one ligand, but recognize multiple molecular structures of both exogenous PAMPs (e.g., LPS, peptidoglycan, and lipoteichoic acid) and endogenous DAMPs (e.g., HMGB1) [15]. Moreover, HMGB1 is the endogenous ligand of all three TLRs [18]. Thus, the transcription and protein expression of these receptors can be influenced by various exogenous and endogenous stimuli, and their modulation depends on miscellaneous influences, including regulatory feedback [28,67,68].

Gram-positive BB12 alone did not upregulate TLR2 expression in the ileum, colon, and mesenteric lymph nodes. However, Gram-negative LT2 upregulated it in the ileum and mesenteric lymph nodes and combination with BB12 in all three observed organs. This finding is seemingly controversial. However, TLR2 recognizes shared patterns of both Gram-positive and Gram-negative bacteria, e.g., lipoproteins [69] and some lipopolysaccharides [67]. Moreover, this signaling pathway uses the CD14 molecule, which is mainly known as a co-receptor of the TLR4/MD-2 signaling pathway [69].

TLR4 was significantly upregulated in all organs of piglets infected with *S*. Typhimurium. The previous association with BB12 alleviated the upregulation in the mesenteric lymph nodes, but not in the intestine. The released LPS is bound to the LPS-binding protein, trapped by the CD14 molecule, and transported to TLR4/MD-2 complex [69]. The activation of the TLR4 pathway depends on the LPS structure and completeness of LPS. LT2 isogenic Δ*rfa* mutants with truncated R-LPS were shown to be less effective in the activation of TLR4/MD-2 signaling pathway and induction of local and systemic inflammatory cytokine levels than wild-type LT2 with S-LPS [53]. While released LPS causes life-threatening endotoxin shock [70], non-typhoidal avirulent *Salmonella* serovars with R-LPS can induce an inflammatory reaction that protects GN piglets against the subsequent infection with S-LPS virulent *S*. Typhimurium [71,72,73].

TLR4 is only one of the TLRs that use both MyD88 and TRIF adaptor molecules in cell surface and endosomal TLR4 signaling, respectively [67]. MyD88-dependent and TRIF-dependent signaling consequent in different spectrums of produced inflammatory cytokines [13]. Similar profiles of TLR4 and MyD88 in the piglet groups in the ileum and mesenteric lymph nodes attest to MyD88 as the main adaptor molecule mediated inflammatory signaling in the *Salmonella* infection [74]. In contrast, this trend did not appear in the colon, and the downregulation of TRIF in *Salmonella* infection was obvious. Similar trends in preterm groups infected with *S*. Typhimurium were observed in the ileum, colon, and mesenteric lymph nodes in term GN piglets, independent of previous association with pig commensal *Lactobacillus amylovorus*, *Lactobacillus mucosae*, or probiotic *Escherichia coli* Nissle 1917 [75]. In contrast to direct contact with *Salmonella* and other bacteria with host intestinal tissue, *Salmonella* only translocated to mesenteric lymph nodes in GN piglets associated with mucinolytic *B. boum* [53,61].

### 3.5. Cytokines

Monocytes/macrophages and neutrophil granulocytes are the first-line sentinel cells of the innate immune response that are early prenatally developed [76,77,78]. Thus, a broad spectrum of inflammatory mechanisms is available for a non-specific immune response immediately after birth. However, their excessive production is known as a “cytokine storm” [79] and can cause multiple organ dysfunction [80]. Possible discrimination between physiological and pathological levels predetermines inflammatory cytokines as members of sepsis biomarkers [81]. Commonly used interleukins (IL)-8, IL-10, and tumor necrosis factor (TNF)-α were also found to be valuable markers of enteric infections in GN piglets [82], and higher levels in intestinal tissue and plasma were found in *Salmonella*-infected preterm GN piglets [46]. IL-6 and IL-12/23p40 are other cytokines that go together with IL-8, IL-10, and TNF-α as biomarkers of prenatal and postnatal inflammation [83,84] and neonatal sepsis [85,86,87,88].

We found that infection with *Salmonella* excessively upregulated ileal, colonic, and plasma IL-6 and IL-12/23/p40 levels within the acute phase of the immune response. Their excessive levels attest to the deleterious effect of the *Salmonella* infection on the immunocompromised preterm GN piglets. The previous colonization of piglets with BB12 prevented a significant increase in IL-6 and IL-12/23p40 intestinal and plasma levels after infection with *Salmonella* compared to control GF piglets. BB12 ameliorated the cytokine storm [79] and the subsequent multiple dysfunction syndrome as its consequences [80].

### 3.6. HMGB1 Protein Expression in the Ileum and Its Intestinal and Plasmatic Levels

HMGB1 is a DNA-binding nuclear protein crucial for transcription that orchestrates responses to tissue damage and repair [17]. Released HMGB1 is also an inflammatory mediator with cytokine activity that emphasizes the production of inflammatory cytokines of intestinal inflammation associated with endotoxemia and NEC [89]. Intestinal HMGB1 was described as an inflammatory bowel disease (IBD) marker in children and a marker of the severity of enteric infections in GN piglets [90]. The part of GN piglets infected with necrotoxigenic *E. coli* O55 that relatively thrived showed low levels of plasmatic and intestinal HMGB1, but the piglets that suffered from the infection showed highly increased levels [91]. Our present finding confirms HMGB1 participation in a cytokine storm [79] with its detrimental effect in the *Salmonella*-infected GN piglets.

HMGB1 levels can be increased by the active secretion of immune cells or its passive release from necrotic cells [17]. Previously, we showed histopathological changes in the intestine in the *Salmonella*-infected preterm piglets [46]. In this work, we presented changes in the localization of HMGB1 in the enterocytes, justifying that increased levels in the *Salmonella*-infected piglets originated from both its stimulated secretion and necrotic release.

### 3.7. Conclusions

Exaggerated levels of cytokines within a cytokine storm [79] have systemic effects due to the damage of vital organs [92]. A modification of microbiota and renewal of its balance can be a therapeutic way of preventing or modulation of MOD in sepsis-suffered patients and increasing the ratio of patients that thrive [93]. Mono-associated GN piglets are the first step of bacterial interference studies and their consequences for the immunocompromised host. The GN piglets associated with a defined synthetic microbiota will be the logical next step in our future research.

## 4. Materials and Methods

### 4.1. Bacteria

*Bifidobacterium animalis* subsp. *lactis* BB-12 (BB12) was isolated from a commercial preparation Biopron Respiron (Valosun, Trinec, Czechia) on modified Wilkins–Chalgren agar (Oxoid, Basingstoke, UK) supplemented with soya peptone (5 g/L; Oxoid), mupirocin (100 mg/L), and acetic acid (1 mL/L) in anaerobic jars with AnaeroGen sachets (Oxoid) and incubated at 37 °C for 48 h, as we described elsewhere [46]. *Salmonella enterica* serovar Typhimurium strain LT2 (*S*. Typhimurium or LT2) [77] was from a collection of microorganisms from the Institute of Microbiology of the Czech Academy of Sciences (Novy Hradek, Czechia). It was cultivated on meat-peptone agar slopes (blood agar base; Oxoid) at 37 °C overnight. Then 8 log CFU/mL BB12 and LT2 suspensions in PBS were prepared for application to animals.

### 4.2. Gnotobiotic Piglets

**Figure 9:**
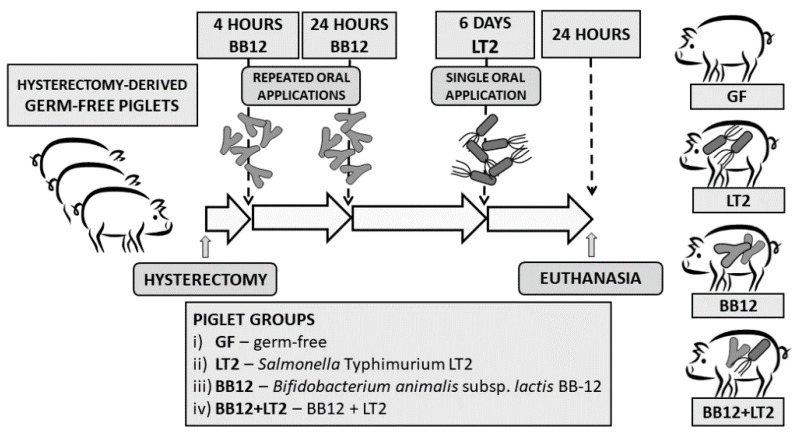
Experiment design. Preterm gnotobiotic piglets (*n* = 24) were assigned into four groups with six piglets per group: (i) germ-free (GF), (ii) infected with *Salmonella* Typhimurium strain LT2 (LT2), (iii) associated with probiotic *Bifidobacterium animalis* subsp. *lactis* BB-12 (BB12), and (iv) associated with BB12 and infected with LT2 (BB12 + LT2).

Preterm miniature germ-free (GF) piglets were derived by hysterectomy on day 104 of pregnancy and reared in fiberglass isolators with a partially heated floor; they were fed 6–7 times per day with cow-milk-based formula. Their microbiological state was tested as described elsewhere [29]. Piglets (*n* = 24) were divided into four groups, with six piglets per group (Figure 9), and orally colonized/infected with BB12 (BB12), LT2 (LT2), and their combination (BB12 + LT2), as we showed (Figure 9) and described previously [46]. The bacteria were orally administered in 5 mL of the milk diet, and the control piglets (GF) received 5 mL of milk without bacteria. At the end of the experiment, the piglets were euthanized by exsanguination via cardiac puncture under isoflurane anesthesia.

### 4.3. Mucin and Goblet Cells in the Ileum and Colon

Acid mucin-producing cell density per area of the tunica mucosa was assessed as described elsewhere [61]. Briefly, Carnoy’s fluid-fixed terminal ileum and colon were dehydrated and embedded in paraffin, and 5 μm cross-sections were stained with Alcian Blue and post-stained with Nuclear Fast Red. The specimens were examined under an Olympus BX 40 microscope with an Olympus Camedia C-2000 digital camera (Olympus, Tokyo, Japan).

### 4.4. Intestinal Lavage and Blood Plasma

Sections (40 cm) of proximal jejunum and the whole ileum with distal jejunum segments were filled with 2 mL of Dulbecco’s PBS (DPBS; TPP, Pasching, Austria), gently kneaded, and rinsed. The colon was cut into small pieces and lavaged in 4 mL of DPBS. The lavages were briefly vortexed and centrifuged at 2500× *g* for 30 min at 8 °C, and supernatants were filtered through a 0.2 μm filter (Sartorius, Goettingen, Germany). Citrated blood was withdrawn by cardiac puncture and centrifuged at 1200× *g* for 10 min at 8 °C. A protease inhibitor cocktail (Roche Diagnostics, Manheim, Germany) was added to the lavage filtrates and plasma, and their aliquots were frozen and stored at −45 °C until processing.

### 4.5. RNA Purification and cDNA Synthesis

The terminal ileum and transverse colon cross-section slices and small pieces of mesenteric lymph nodes were put into RNAlater and stored at −20 °C. Later they were moved into the RTL buffer of RNeasy Mini Kit Plus (Qiagen) and homogenized with 2 mm zirconia beads (BioSpec Products, Bartlesville, OK, USA) in TissueLyser LT beadbeater (Qiagen). The total RNA was isolated according to the manufacturer’s protocol. A total of 500 ng of total RNA was reverse transcribed by QuantiTect Reverse Transcription kit (Qiagen). The prepared cDNA was 1/10 diluted by PCR quality water (Life Technologies, Carlsbad, CA, USA), and this cDNA template was stored at −25 °C till quantitative PCR was performed.

### 4.6. Real-Time PCR

A total of 2 μL of the cDNA template was added into 18 μL of the FastStart Universal Probe Master (Roche Diagnostics), with 500 nM each of forward and reverse primers and 100 nM locked nucleic acid (LNA) probe (Universal ProbeLibrary; Roche Diagnostics). The PCR systems for the reference genes β-actin and cyclophilin A, as well as for the genes of interest, TLR2, TLR4, TLR9, MyD88, and TRIF, were listed elsewhere [29]. The PCR amplification was performed in duplicates in 45 cycles (95 °C for 15 s and 60 °C for 60 s) and run on an iQ cycler (Bio-Rad, Hercules, CA, USA). The evaluation of relative mRNA expression (fold changes) was described elsewhere [46].

### 4.7. Intestinal and Plasmatic HMGB1 Levels

HMGB1 levels in the intestinal lavages (jejunum, ileum, and colon) and blood plasma were measured by ELISA kit (Abbexa, Cambridge, UK), according to the producer’s instructions. The absorbances were measured at 450 and 620 nm on an RS ELISA reader (Labsystems, Helsinki, Finland), and the results were evaluated with Genesis 3 software (Labsystems).

### 4.8. IL-6 and IL-12/23 p40 in Intestinal Lavage and Blood plasma

Levels of IL-6 and IL-12/23 p40 in the intestinal lavages and plasma were measured by a paramagnetic sphere-based xMAP technology (Luminex Corporation, Austin, TX, USA) with a Porcine ProcartaPlex kit (Affymetrix, Santa Clara, CA, USA) on the Bio-Plex Multi Array System (Bio-Rad, Hercules, TX, USA) and evaluated by Bio-Plex Manager 4.01 software (Bio-Rad), as described previously [75].

### 4.9. Immunochemical Detection of HMGB1 in the Colon

The transverse colon was embedded in Tissue-Tek (Sakura, Tokyo, Japan), immediately frozen in liquid nitrogen vapor-cooled isopentane, and kept at −70 °C. Then 5 μm acetone-fixed cryosections on SuperFrost/Plus slides (Thermo Fisher Scientific, Darmstadt, Germany) were stored at −40 °C until labeling. After the incubation of sections with 5% goat serum (Life Technologies, Carlsbad, CA, USA) for 1 h at RT, they were labeled by anti-HMGB1 rabbit polyclonal antibodies (Novus Biologicals, Centennial, CO, USA) overnight, at 4 °C. The sections were incubated with a peroxidase-conjugated goat anti-rabbit F(ab)2 IgG fragment (Invitrogen, Carlsbad, CA, USA) for 2 h at RT. HMGB1 was visualized by AEC substrate (Sigma-Aldrich, St. Louis, MO, USA), and nuclei were counterstained with Mayer’s hematoxylin (Diapath, Martinengo, Italy). Control sections without primary antibodies were treated in the same way. The sections were examined under an Olympus BX 40 microscope with Olympus Camedia C-2000 digital camera (Olympus, Tokyo, Japan), as described elsewhere [94].

### 4.10. Statistical Analysis

Normally distributed values were compared with two-way analysis of variance (ANOVA) with Tukey’s multiple comparisons post hoc test. Values that did not meet the normal distribution were evaluated with Kruskal–Wallis with Dunn’s multiple comparisons post hoc test. The statistical comparisons were performed at *p* < 0.05 by GraphPad 6 software (GraphPad Software, San Diego, CA, USA), and significant differences were depicted in figures by a letter system.

## Data Availability

Data are available upon request from the corresponding author.
